# The Biosynthetic Pathway of Mycolic Acids: Dual-Function Targets for Tuberculosis Therapeutics and Green Steroid Drugs Biomanufacturing

**DOI:** 10.3390/pharmaceutics18010044

**Published:** 2025-12-29

**Authors:** Yupan Zhou, Xianya Wang, Wanting Jia, Zhengding Su, Xiyao Cheng

**Affiliations:** 1 Institute of Modern Fermentation Engineering and Future Foods, School of Light Industry and Food Engineering, Guangxi University, No. 100, Daxuedong Road, Nanning 530004, China; 2316302017@st.gxu.edu.cn (Y.Z.); 2316392016@st.gxu.edu.cn (X.W.); 2316393001@st.gxu.edu.cn (W.J.); 2 School of Pharmaceutical Sciences and Institute of Materia Medica, Xinjiang University, Urumqi 830017, China; james_su@xju.edu.cn

**Keywords:** mycolic acids (MA), *Mycobacterium*, biosynthesis regulation, anti-tuberculosis drugs

## Abstract

Mycolic acids (MAs) are unique and essential components of the *Mycobacterium* cell envelope, pivotal for its structural integrity, impermeability, and intrinsic antibiotic resistance. These properties that underpin mycobacterial pathogenicity also render the MA biosynthetic pathway a rich resource of targets for anti-tuberculosis drug discovery. Concurrently, in the realm of industrial biotechnology, engineered non-pathogenic mycobacteria are being optimized for steroid drug bioproduction through strategic modulation of the MA pathway to enhance cell permeability and boost the yield of desired products. This review systematically delineates the MA biosynthetic pathway and its critical enzymes. It further summarizes recent progress in developing anti-tuberculosis therapeutics that inhibit these enzymes and discusses innovative engineering strategies that harness the same pathway of non-pathogenic mycobacteria for green steroid drug manufacturing. By bridging these two distinct fields, the review provides a holistic perspective and novel insights for advancing both infectious disease control and sustainable pharmaceutical production.

## 1. Introduction

*Mycobacterium* species are Gram-positive, aerobic, rod-shaped bacteria found in diverse environments, including soil, water, and host cells. Based on pathogenicity, these species are divided into pathogenic and non-pathogenic groups [1]. Among the pathogenic mycobacteria, *Mycobacterium tuberculosis* (*M. tuberculosis*) is the primary etiological agent of human tuberculosis (TB). In contrast, non-pathogenic species such as *Mycobacterium smegmatis* (*M. smegmatis*) and *Mycobacterium neoaurum* (*M. neoaurum*) are characterized by distinct metabolic pathways and unique cell wall structures, which render them particularly suitable as hosts for the efficient biotransformation of steroid compounds, offering distinct advantages in industrial applications.

*M. tuberculosis* was the foremost killer among single infectious agents until the COVID-19 pandemic, with mortality and infectivity rates exceeding those of HIV/AIDS [2]. Isoniazid (INH), a cornerstone anti-tuberculosis drug, exerts its bactericidal effect by inhibiting InhA, a key enzyme in MA biosynthesis [3]. However, the efficacy of conventional treatments has been significantly compromised by the emergence and spread of multidrug-resistant (MDR-TB) and extensively drug-resistant (XDR-TB) strains, posing a global health challenge. Addressing this, newer drugs like Delamanid and Pretomanid act on MA synthesis while also releasing nitric oxide to disrupt the electron transport chain and redox balance, thereby providing innovative mechanisms to combat resistance [4,5]. Consequently, elucidating the role of MA is fundamental to advancing our understanding of TB pathogenesis and to laying the groundwork for more effective and less toxic next-generation drugs.

Non-pathogenic mycobacteria, particularly *M. neoaurum*, have become key microbial hosts for the industrial production of steroid drug intermediates due to their unique capacity to efficiently cleave phytosterol side chains [6]. These strains selectively synthesize critical steroid intermediates such as 9α-hydroxyandrost-4-ene-3,17-dione (9-OHAD), 4-androstene-3,17-dione (4-AD), and 22-hydroxy-23,24-bisnorchol-4-ene-3-one (4-HBC), which are essential precursors for the synthesis of sex hormones, progestogens, and corticosteroids. However, the industrial application of *M. neoaurum* remains constrained by suboptimal conversion efficiency. Recent advances in genetic and metabolic engineering, combined with multi-omics analyses, have enabled targeted modifications of the *M. neoaurum* cell wall, particularly through the regulation of MA biosynthesis, leading to significant improvements in conversion performance. For instance, knockout of genes such as *MmpL3* [7], *KasB* [8], and *fbpC3* [9] has been shown to enhance cell membrane permeability, facilitating the uptake of sterol substrates and promoting the accumulation of target products. These developments provide a crucial technological foundation for the efficient, green, and sustainable biomanufacturing of steroid drugs, demonstrating considerable industrial potential and promising future prospects.

To systematically review the development of this field, we searched core databases such as Web of Science and IEEE Xplore using “[Mycolic acids]” and “[*Mycobacterium*]” as primary keywords. The search encompassed relevant literature up to 2025. We selected high-impact studies directly related to the topic and traced the citation networks of key papers. Based on this analysis, this review systematically outlines the MA biosynthetic pathway, summarizes current anti-tuberculosis drugs targeting it, and presents metabolic engineering strategies for sterol drug bioconversion via MA pathway modification. By integrating these advances, we aim to bridge theoretical knowledge and practical applications, providing valuable insights for future *Mycobacterium* research.

## 2. The Structure and Biological Function of MA in Mycobacteria

### 2.1. Structure and Classification of MA

**Figure 1:**
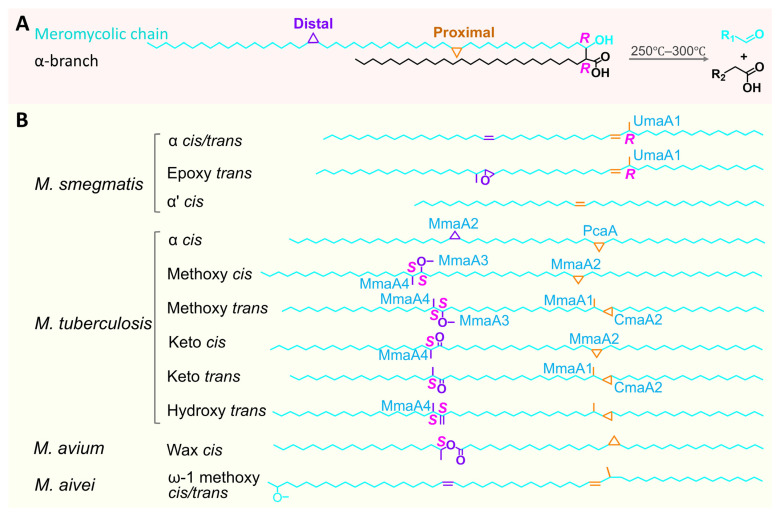
MA Structure and Classification in *Mycobacterium*. (**A**). MA consists of a meromycolic main chain (R1) and an α-branch (R2). (**B**). Variations in meromycolic chain modifications among *Mycobacterium* species.

MA is a long-chain fatty acid with α-alkyl and β-hydroxy groups, consisting of two main components: a short α-branch at the carboxyl α-position and the longer meromycolic chain extending from the terminal methyl group to the hydroxyl-bearing carbon (Figure 1A) [10]. Additionally, both stereocenters adjacent to the carboxyl group (at the C2 and C3 positions) exhibit an *R*-configuration, a stereochemical feature conserved across all known MAs [11].

As shown in Figure 1A, MA molecules in *Mycobacterium* typically contain 60–90 carbon atoms, with some species exceeding 100. Marrakchi et al. indicate that the α-branch is a fixed-length, saturated structure, usually composed of 20, 22, or 24 carbon atoms [12]. MA chain length influences membrane fluidity: longer chains reduce fluidity [13]. Modifications to the meromycolic chain primarily occur at the proximal and distal ends near the β-hydroxy group. The proximal region often features hydrophobic modifications, including *cis*-cyclopropanation, double bond formation, or methylation. The distal region typically exhibits cyclopropane rings or oxidative modifications, such as keto-, epoxy-, and hydroxy-groups, as well as methoxy groups and wax esters (Figure 1B). Barry observed that these oxidative modifications are frequently accompanied by adjacent methyl branches [14], further increasing the structural diversity of MA. MA can be classified into α-MA, methoxy-MA, and keto-MA types based on differences in distal functional groups. Typically, α-MA contains only *cis*-cyclopropane modifications, while methoxy-MA and keto-MA may exist in both *cis* and *trans* isomers, depending on the bacterial strain. Laval et al. reported that oxygenated MAs in *M. tuberculosis* generally have carbon chains ranging from 84 to 88, 4–6 carbons longer than the α-MA in the same strain [15,16]. In certain non-tuberculous mycobacteria, epoxy-MA and wax ester-MA exhibit chain lengths similar to α-MA.

The variation and relative abundance of MAs significantly influence the physicochemical and biological properties of the cell wall. For example, a higher proportion of α-MA increases membrane thickness, while elevated levels of keto-MA and methoxy-MA reduce outer membrane thickness but enhance stability [17,18]. Oxygenated MAs are crucial for *M. tuberculosis* virulence. Specifically, the loss of methoxy-MA impairs biofilm formation [19], with biofilm thickness positively correlating with virulence and drug resistance. Additionally, Capua and colleagues found that the absence of α’-MA was shown to compromise antibiotic resistance in non-tuberculous mycobacteria [20].

### 2.2. Functions of Different Classes of MA in the Cell Wall

**Figure 2:**
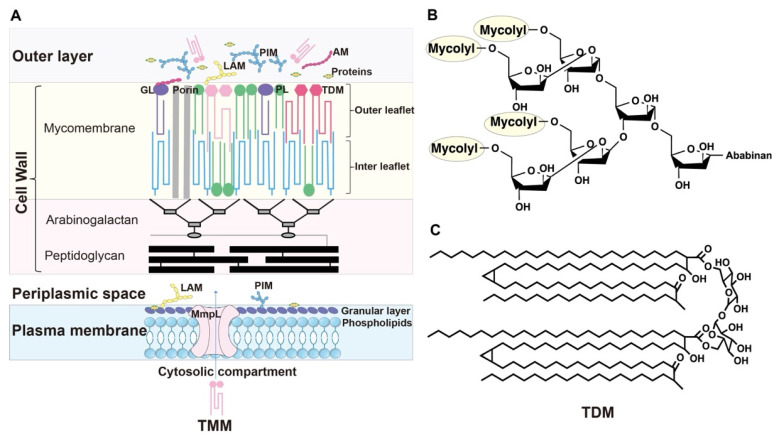
Composition of the Mycobacterial Cell Envelope. (**A**). Structure map of the Mycobacterial cell Envelope. The mycobacterial envelope comprises the outermost layer, cell wall, and plasma membrane. The cell wall consists of the mycomembrane, arabinogalactan (AG) layer, and peptidoglycan (PG) layer. The MA chain (blue) in the interleaflet region is linked to AG, which is covalently bonded to PG, forming the mAGP complex (MA–AG–PG). (**B**). The hexa-arabinofuranoside motifs conjugated to MA in the mAGP complex. (**C**). Molecular structure of trehalose dimycolate (TDM).

MA, the principal component of the mycomembrane [21], is a hydrophobic long-chain fatty acid that forms a dense barrier, reducing membrane permeability and blocking the entry of antibiotics, thereby imparting resistance to extreme environments and contributing to multidrug resistance [22]. In *Mycobacterium*, MAs primarily exist in three classes (Figure 2A).

The first class of MA is oriented perpendicular to the cell wall, densely packed within the inner leaflet of the cell membrane [23]. Approximately two-thirds of the MA molecules cluster in groups of four, binding to the C5 hydroxyl groups on the terminal hexaarabinofuranoside of the non-reducing end of AG via β-1,4 glycosidic bonds (Figure 2B). AG is covalently linked to PG, forming the mAGP complex, which restricts lateral mobility of MA molecules. This structure imparts unique conformational flexibility to the “head groups” of hexaarabinofuranoside, contributing to the permeability barrier and drug resistance of *Mycobacterium* [23]. Savintseva et al. found that the predominant *trans* “W-shaped” conformation in the inner leaflet reduces membrane fluidity and permeability, while enhancing drug resistance and virulence [18].

The second class of MA is located in the outer leaflet of the mycomembrane, where two molecules of trehalose monomycolate (TMM) are esterified to form trehalose dimycolate (TDM) (Figure 2C), which play critical roles in immune regulation and mycobacterial virulence [24]. TDM activates immune receptors, such as macrophage-inducible C-type lectin (Mincle) [25], triggering the production of inflammatory mediators like nitric oxide (NO) and tumor necrosis factor (TNF), thereby enhancing immune responses [26]. TDM is central to *M. tuberculosis* pathogenesis, inhibiting phagosome maturation in macrophages and aiding immune evasion [26]. Additionally, TDM modulates granuloma formation by altering cholesterol levels within macrophages [27]. As shown in Figure 2C, TDM contains exceptionally long MA chains that fold at specific units—such as cyclopropane rings and keto groups—before ordered stacking. This folding is critical for maintaining the typical membrane thickness of 7–8 nm. Together, the first two classes of MA constitute up to 50% of the mycobacterial cell wall’s dry weight, contributing to its low permeability.

The third class of MA is secreted in a free form extracellularly [21] and plays a key role in *M. tuberculosis* infection. One of its major functions is promoting biofilm formation, which enhances antibiotic resistance [28]. Under specific in vitro conditions, *M. tuberculosis* produces biofilms enriched in free MA, primarily released from TDM via hydrolysis by serine esterases [29]. In addition to facilitating biofilm formation, free MAs modulate host immune responses by suppressing pro-inflammatory pathways, thereby aiding bacterial survival under immune stress [30]. They also interact with intracellular pattern recognition receptors, such as NOD-like receptors (NLRs), to help maintain a balanced inflammatory response [27,31]. Furthermore, free MA may promote nutrient acquisition, supporting bacterial survival during host-induced nutrient starvation [32].

## 3. Biosynthetic Pathway of MA and Associated Key Enzymes

**Figure 3:**
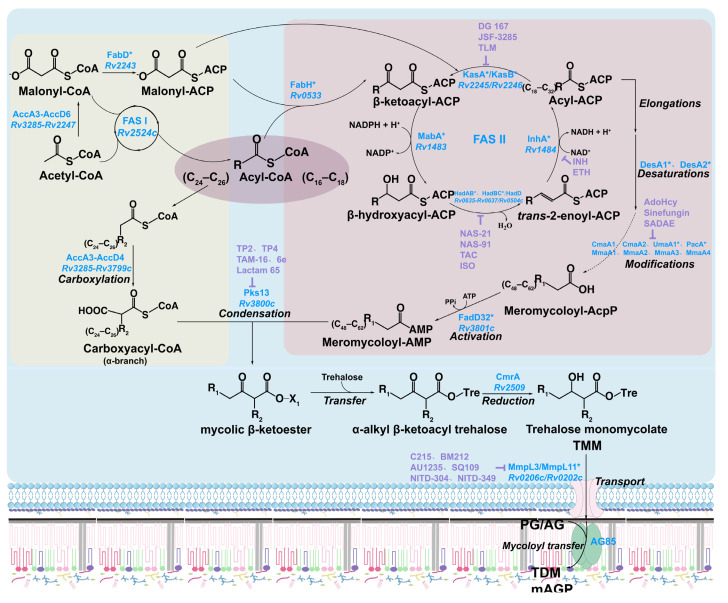
Biosynthetic Pathway of MA in *Mycobacteria*. During MA biosynthesis, the FAS-I (yellow module) and FAS-II (pink module) fatty acid synthase systems synergistically generate the α-branch and meromycolic chain. Then, these precursors condense to form MA, which is subsequently transported to the outer membrane via a transmembrane system. Enzymes marked with “*” are regulated at the transcriptional and post-translational levels via Ser/Thr phosphorylation. Inhibitors targeting enzymes in the MA biosynthesis pathway are highlighted in purple.

The biosynthesis of MA in *M. tuberculosis* relies on two distinct fatty acid (FA) synthase systems: FAS-I and FAS-II [12]. FAS-I synthesizes the α-branch, while FAS-II elongates the meromycolic chain. Ultimately, these components are joined to form MA (Figure 3). FAS-I is a large, multifunctional enzyme complex found in higher prokaryotes, eukaryotes, and animals. It catalyzes the de novo synthesis of fatty acids (FAs) from acetyl-CoA and malonyl-CoA, producing chains of two primary lengths: C_16_–C_18_ and C_24_–C_26_. The longer C_24_–C_26_ FAs serve as precursors for the α-branch of MA. FAS-II is a modular system of soluble enzymes found in plants and bacteria that elongates C_16_–C_18_ FAs produced by FAS-I through iterative two-carbon extensions using malonyl-CoA, ultimately forming ultralong meromycolic chains. The α-branch and meromycolic chain are then joined by a Claisen-type condensation reaction to yield MA structure.

### 3.1. Synthesis of Malonyl-CoA

**Figure 4:**
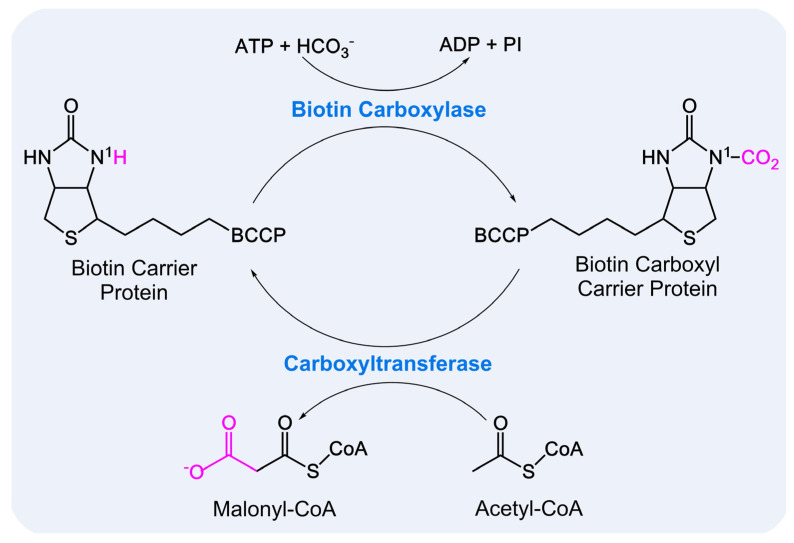
Biotin-Dependent Carboxylation Reaction in ACCase.

Malonyl-CoA serves as a universal two-carbon donor for the synthesis of MAs and other FAs. It is produced from acetyl-CoA through a biotin-dependent, two-step reaction catalyzed by acetyl-CoA carboxylase (ACCase) and participates in the catalytic cycles of FAS I/FAS II [33]. In mycobacteria, ACCases are multi-subunit complexes, typically composed of at least one α subunit (AccA), which includes the biotin carboxylase (BC) and biotin carboxyl carrier protein (BCCP) domains, and one β subunit (AccD), containing the carboxyltransferase (CT) domain [34,35]. Biotin-dependent carboxylation proceeds in two steps: first, the BC domain couples carbonate to the N(1) position of the biotin ureido ring attached to the BCCP, forming carboxybiotin; second, the CT domain transfers the carboxyl group from biotin to acyl-CoA, yielding carboxylated acyl-CoA [33], as shown in Figure 4. In *M. tuberculosis*, ACCases comprise three α subunits (AccA1–AccA3), six β subunits (AccD1–AccD6), and one ε subunit (AccE) [36]. The β subunits determine substrate specificity, enabling ACCases to carboxylate various substrates, including acetyl-CoA and propionyl-CoA.

### 3.2. Synthesis of the α-Branch

*Mycobacteria* utilize the multifunctional enzyme FAS-I to synthesize FA de novo, initiating from acetyl-CoA and using malonyl-CoA for chain elongation. This process generates a bimodal distribution of long-chain acyl-CoA: C_16_–C_18_ acyl-CoA, primarily used for membrane phospholipid synthesis, and C_24_–C_26_ acyl-CoA, which is carboxylated by the AccA3-AccD4 complex to form the α-branch [37]. FAS-I is an essential, multifunctional enzyme encoded by the *fasI* gene (*Rv2524c*) in *Mycobacterium*, containing catalytic domains for acyltransferase, enoyl reductase, dehydratase, malonyl/palmitoyl transferase, acyl carrier protein, ketoreductase, and ketoacyl synthase [38]. FAS-I is crucial for the survival of *M. tuberculosis* and *M. smegmatis* [39]. In *M. smegmatis*, reduced FAS-I expression leads to the accumulation of acetyl-CoA and malonyl-CoA, disrupting the synthesis of short- and medium-chain FA and altering the relative abundance of α-MA, α’-MA, and epoxy-MA.

### 3.3. Synthesis of the Meromycolic Backbone

**Figure 5:**
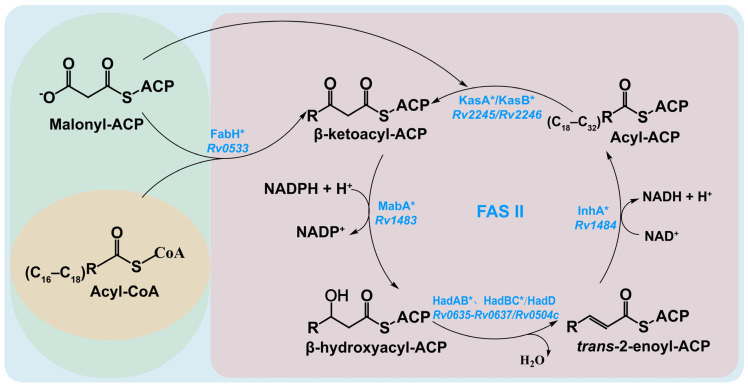
FAS-II System-Mediated MA Backbone Synthesis Pathway. Enzymes marked with “*” are regulated at the transcriptional and post-translational levels via Ser/Thr phosphorylation.

The FAS-I and FAS-II systems are interconnected via β-ketoacyl-ACP synthase III (FabH), which preferentially uses C_16_–C_18_ acyl-CoA as a substrate, facilitated by its conserved catalytic triad and hydrophobic pocket. FabH condenses this substrate with malonyl-ACP, produced by malonyl-CoA: ACP transacylase (FabD) from the FAS-I system, to form β-ketoacyl-ACP [40]. This intermediate then enters the FAS-II cycle for chain elongation (Figure 5). The β-ketoacyl-ACP is processed sequentially by NADP-dependent β-ketoacyl-ACP reductase (MabA) [41], β-hydroxyacyl-ACP dehydratases (HadAB, HadBC, HadBD) [42,43], NADH-dependent trans-2-enoyl-ACP reductase (InhA) [44], and β-ketoacyl-ACP synthases (KasA/KasB) [45], extending the acyl-ACP chain to lengths required for meromycolic chain modification. Meanwhile, the FAS-I system continuously supplies malonyl-ACP to sustain FAS-II elongation. InhA, MabA, KasA, and HadB are essential for MA biosynthesis, while HadA, HadC, HadD, and KasB are non-essential [43,46,47,48].

### 3.4. Modification of the Meromycolic Chain

During meromycolic chain synthesis, two *cis* double bonds are introduced at the distal and proximal ends via the desaturation of the initially saturated alkyl chain. Experimental data from Barry indicate that desaturation typically occurs before α-branch attachment and is closely coupled with chain elongation [14]. In *M. tuberculosis*, desaturation involves desaturases such as DesA1 [49] and DesA2 [50], along with (*R*)-3-hydroxyacyl-CoA dehydratase FabQ, which has combined dehydration and isomerase activity [51]. Lanéelle et al. suggest that β-ketoacyl-ACP synthases (KasA/KasB) catalyze decarboxylation and condensation reactions, sequentially linking FAs and determining the positions of double bonds and oxidative modifications [52].

The unsaturated meromycolic chain undergoes further modifications, including cyclopropanation, methylation, and oxidation, to form meromycolyl-AcpM [53]. These processes are catalyzed by a family of S-adenosylmethionine-dependent methyltransferases (SAM-MTs) [54]. The mycobacterial MT family consists of eight enzymes: PcaA (also known as UmaA2), UmaA1, MmaA1, MmaA2, MmaA3, MmaA4, CmaA1, and CmaA2 [55]. Cyclopropanation is primarily mediated by PcaA, UmaA1, MmaA1, and MmaA2 [56,57], which introduce cyclopropane rings at unsaturated bonds in the MA chain. MmaA1, MmaA4, and UmaA1 directly facilitate cyclopropane formation and, through methylation, introduce methyl branches at adjacent positions on the unsaturated bonds or cyclopropane ring [58,59].

Among the oxidative modifications of MA chains in mycobacteria, hydroxy-MA serves as a key intermediate, produced through MmaA4-catalyzed modification of a distal double bond [60]. Hydroxy-MA is subsequently converted to methoxy-MA by MmaA3 [61], while the oxidase responsible for converting hydroxy-MA to keto-MA remains unidentified. Additional enzymes involved in oxidative modifications include the epoxy hydrolase EphD and β-hydroxyacyl-ACP dehydratase HadD, which contribute to keto-MA and methoxy-MA biosynthesis [62,63]. Capua et al. identified that the *MSMEG_1350* gene encodes a methyltransferase involved in the synthesis of epoxy-MA [64].

### 3.5. Condensation and Transfer of MA

After modification by FAS-II, meromycolyl-AcpM is activated to meromycolyl-AMP by the fatty acyl-AMP ligase FadD32 [65]. Meromycolyl-AMP then undergoes a Claisen condensation with the α-branch, forming an α-alkyl, β-ketoacyl intermediate [66]. This intermediate reacts with trehalose, which is transported into the membrane by the secretory protein Proline-Proline-Glutamic acid (PPE51), resulting in α-alkyl, β-ketoacyl trehalose [67]. The MA reductase CmrA then reduces this product to trehalose TMM [67,68].

TMM molecules are transported to the periplasm by the membrane transporter MmpL3, which utilizes a flippase-like mechanism via its transmembrane helices [69]. Assisted by molecular chaperones, TMM then crosses the periplasm space to reach the mycomembrane, where the acyltransferase Antigen 85 (Ag85) complex transfers a MA chain from one TMM molecule to another, forming TDM, or TMM covalently binds to arabinogalactan to form the mAGP complex. This allows MA to be incorporated into the cell wall in the forms of TMM, TDM, and mAGP complexes [70].

## 4. Regulation of Mycobacterial MA Biosynthesis in Native Environments

MA is a critical structural component maintaining the *Mycobacterium* cell wall [53]. Growth of *Mycobacterium* depends on the ongoing synthesis and expansion of the cell wall, which requires precise temporal and spatial coordination of cell elongation, chromosome replication and segregation, and cell division. This process also adapts to environmental changes, balancing energy consumption with lipid synthesis. To orchestrate these processes, *Mycobacterium* has developed multi-level regulatory mechanisms for MA biosynthesis. Current research on endogenous regulation of MA synthesis focuses on two main aspects: transcriptional control of key biosynthetic enzymes and post-translational modifications.

### 4.1. Transcriptional Regulation of Key MA Synthesis Enzymes

In mycobacteria, the biosynthesis of MA, from FA synthesis to meromycolic chain desaturation, is primarily regulated by three transcription factors: FasR, MabR, and MadR [71,72,73]. FasR (*Rv3208*) is a transcriptional activator that regulates the *fas-acpS* operon, involved in the FAS-I system of MA biosynthesis. Its activity is inhibited by long-chain acyl-CoA (C_16_ and longer), providing feedback control to limit FAS-I product accumulation [74].

MabR (*Rv2242*), a member of the PucR transcription factor family [75], is located upstream of the *fas-II* operon and its binding activity is regulated by C_18_–C_24_ acyl-CoA chains [76]. Initial studies indicated MabR acts as a transcriptional repressor, with Salzman et al. showing that its overexpression in *M. smegmatis* inhibited the *fas-II* operon, reducing FA synthesis [73]. However, later work by Tsai et al. revealed that low-level MabR expression downregulated *fas-II* transcription, suggesting MabR may function as an activator of the *fas-II* gene cluster under physiological conditions [76].

MadR (*Rv0472c*), a member of the TetR family, is a key repressor of MA biosynthesis, controlling the expression of desaturases DesA1 and DesA2 [77]. Under normal conditions, MadR represses their expression by binding to the promoters. However, in response to membrane stress or altered lipid metabolism, this repression is relieved, leading to the upregulation of DesA1/DesA2 and increased MA desaturation [49,72]. MadR binds a range of acyl-CoA molecules, but only dissociates from the promoters when bound to saturated acyl-CoA (C_16_ or longer), enabling metabolic sensing regulation of MA synthesis [76]. Deletion of MadR results in excessive DesA1/DesA2 expression, causing aberrant desaturation, accumulation of α-MA, increased membrane permeability, and heightened antibiotic sensitivity [72].

Together, FasR, MabR, and MadR integrate the regulation of FAS-I, FAS-II, and meromycolic chain desaturation in response to fluctuations in endogenous long-chain acyl-CoA. This coordinated regulatory network ensures the dynamic balance of membrane and cell wall structures, enabling mycobacteria to adapt to environmental stress and infection. Notably, MadR serves as a key checkpoint in lipid metabolism, regulating MA biosynthesis and offering potential as a drug target.

### 4.2. Post-Translational Regulation of Key MA Synthesis Enzymes via Serine/Threonine Phosphorylation

In mycobacteria, post-translational modifications, particularly phosphorylation, modulate the activity and stability of key enzymes in the MA biosynthesis pathway. This mechanism enables rapid and efficient adaptation of bacterial growth and virulence in response to environmental conditions. *M. tuberculosis* utilizes 11 serine/threonine protein kinases (STPKs), including PknA, PknB, through PknL, along with the phosphatase PstP, to form a phosphorylation signaling network that governs processes such as central metabolism, cell division, and cell wall biosynthesis [78]. PknA and PknB are essential for bacterial growth and division, modulating cell morphology by phosphorylating FAS-II system enzymes and the peptidoglycan protein CwlM [79].

Key FAS-II enzymes—including KasA/KasB [80], FabH [81], MabA [82], InhA [83], HadAB, and HadBC [84]—are regulated by STPKs, with phosphorylation reducing enzymatic activity. For example, phosphorylation of FabH, MabA, and InhA impairs FA and MA synthesis, while phosphorylation of KasB alters MA chain length and affects virulence [80]. Slama et al. found that during active growth, low phosphorylation favors enzyme activity, whereas in stationary phase, increased phosphorylation of HadAB/BC suppresses MA synthesis, aiding environmental adaptation [84]. PknF regulates cell division and glucose transport [85], while PknG and PknH influence virulence and host adaptation [86,87]. Furthermore, PknK phosphorylates the transcription factor VirS to enhance *mymA* operon expression under acidic conditions, adjusting MA content and cell wall composition [88]. PknL participates in regulating cell wall synthesis and division [89]. The phosphatase PstP counteracts phosphorylation, restoring enzyme activity. PstP itself is phosphorylated at multiple sites and can dephosphorylate cell wall substrates, refining their activity [90].

Phosphorylation of FadD32, a key enzyme in MA precursor synthesis, inhibits its activity, thus limiting MA biosynthesis [91]. Le et al. found that this inhibition is reversed by PstP, maintaining dynamic balance. Similarly, phosphorylation of PcaA suppresses MA cyclopropanation, influencing MA composition and mycobacterial growth [92]. The phosphorylation of the MA transporter MmpL11 regulates its stability, with phosphorylation at T765 modulating protein degradation and controlling transporter activity [93].

## 5. Anti-Tuberculosis Drugs and Metabolic Engineering Approaches Targeting MA Biosynthesis

Mycobacteria depend on MA for cell wall integrity. In *M. tuberculosis*, inhibition of key enzymes in MA biosynthesis exerts antibacterial effects, aiding the development of anti-tuberculosis therapies. In non-pathogenic engineered mycobacterial strains, modulating MA biosynthesis enzymes enhances cell permeability and improves the conversion efficiency of target compounds.

### 5.1. MA Biosynthesis Enzymes as Key Targets for Antituberculosis Therapeutics

Current treatment for drug-susceptible tuberculosis involves a standard six-month regimen: two months of intensive phase using isoniazid (INH), rifampicin (RIF), pyrazinamide (PZA), and ethambutol (EMB), followed by a four-month continuation with INH and RIF [94]. However, the extended treatment duration, high pill burden, and adverse effects often lead to poor patient adherence, contributing to the emergence of multidrug-resistant (MDR), extensively drug-resistant (XDR), and totally drug-resistant (TDR) strains [95]. This highlights the urgent need for new anti-tuberculosis agents with novel mechanisms, enhanced efficacy against resistant strains, and shorter treatment regimens. Enzymes involved in the MA biosynthesis are validated targets for anti-tuberculosis drugs, including INH, ethionamide (ETH), and thiacetazone (TAC) [96].

#### 5.1.1. Anti-Tuberculosis Drugs Targeting 2-trans-Enoyl-ACP Reductase (InhA)

**Figure 6:**
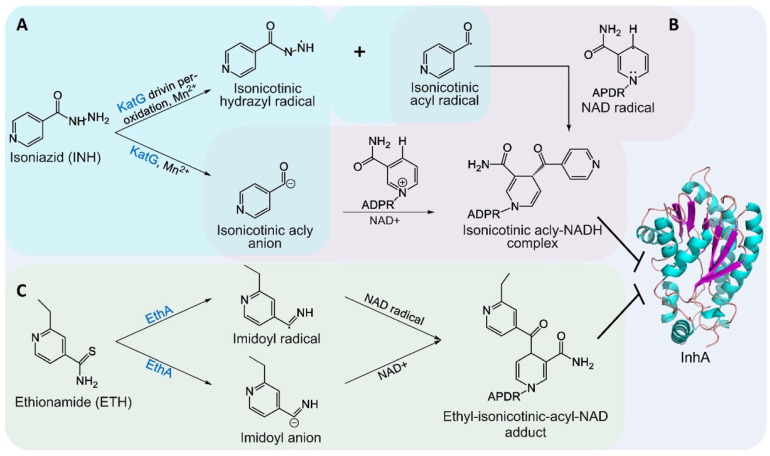
Inhibition of InhA by INH and ETH. (**A**). INH is activated by KatG, generating reactive isoniazid and isonicotinoyl radicals. (**B**). These radicals interact with NAD^+^ at the InhA active site, forming a covalent INH-NADH adduct that inhibits InhA, disrupting meromycolic acid chain elongation. (**C**). ETH is activated by EthA to form a reactive radical, which binds NAD^+^, producing an ETH-NAD adduct that inhibits InhA (PDB ID: 5W07). APDR: NAD side chain.

INH has been a first-line anti-tuberculosis agent since its FDA approval in 1953. Intracellularly, INH is activated by the catalase-peroxidase enzyme encoded by *katG*, generating reactive radicals that interact with NAD^+^ to form an INH–NADH adduct. This adduct inhibits the NADH-dependent reduction reaction catalyzed by InhA in the FAS-II system, specifically blocking the conversion of trans-2-enoyl-ACP to acyl-ACP. thereby disrupting meromycolic chain elongation and impairing MA biosynthesis (Figure 3 and Figure 6) [97]. Resistance to INH can arise from mutations in *katG* or alterations in InhA expression or structure [98,99].

To address MDR-TB, ETH is utilized as a second-line treatment. ETH is activated by the flavin-dependent monooxygenase EthA, generating reactive species that form an ETH-NAD adduct, which inhibits InhA [100]. ETH can also be modified at the 2-position of its pyridine ring to produce derivatives for developing dual-targeting inhibitors, such as prothionamide (PTH), which not only inhibit InhA but also target decaprenylphosphoryl-β-D-ribose-2-epimerase (DprE1), a key enzyme in arabinogalactan synthesis, thereby synergistically compromising cell wall integrity [96].

Due to the limitations of activation-dependent prodrugs and resistance arising from mutations in activation enzymes, direct InhA inhibitors have emerged as promising alternatives. Several small-molecule inhibitors, including arylamides [101], triclosan [102], diphenyl ether derivatives [103], pyrrolidine carboxamides [104], imidazopiperidines [105], tetrahydropyrans such as 4-hydroxy-2-pyridinones [106], and thiazole derivatives [107], directly bind to key sites on InhA, bypassing the need for enzymatic activation and overcoming resistance due to *katG* mutations [108]. However, challenges remain, including low oral bioavailability, toxicity, and unfavorable pharmacokinetics.

#### 5.1.2. Anti-Tuberculosis Drugs Targeting β-Ketoacyl-ACP Synthase

The β-ketoacyl-ACP synthases, including KasA, KasB, and FabH, are key enzymes in mycobacterial MA biosynthesis. FabH links the FAS-I and FAS-II systems [40], while KasA and KasB are critical for FA chain elongation, adding two carbon atoms to acyl-ACP to form β-ketoacyl-ACP that enters the next elongation cycle. KasA initiates chain extension, extending short-chain FAs to approximately 40 carbons, whereas KasB further elongates them, typically up to 54 carbons, forming FAs characteristic of MA [109]. Bhatt et al.’ studies have demonstrated that deletion of *KasB* in *M. tuberculosis* leads to MA with chain lengths shortened by 4 to 6 carbons, along with the loss of trans-cyclopropanated MA, impairing bacterial growth, increasing cell wall permeability, and reducing virulence [110].

**Figure 7:**
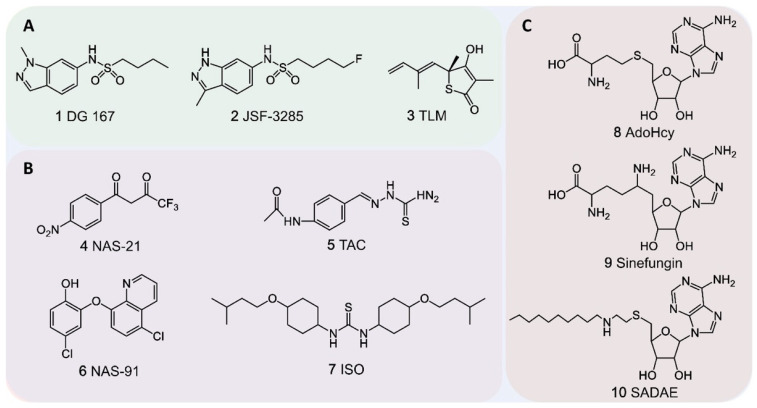
Chemical structures of anti-tuberculosis drugs targeting. (**A**). β-ketoacyl-ACP synthase. (**B**). β-hydroxyacyl-ACP dehydratase. (**C**). The S-adenosylmethionine-dependent methyltransferase family.

Thiolactomycin (TLM) is a broad-spectrum inhibitor of KasA, KasB, and FabH, effectively blocking MA biosynthesis in *M. tuberculosis* [111,112]. The multi-target mechanism of TLM offers several advantages, including broad-spectrum inhibition and low cytotoxicity. This mechanism theoretically increases the genetic barrier to resistance. Moreover, its ‘slow-onset’ inhibition of KasA ensures prolonged target residence time. Despite these favorable pharmacological properties, TLM fails to maintain adequate concentrations at the infection site [113], significantly limiting its anti-tuberculosis efficacy in vivo. Therefore, optimization of the thiolactone scaffold should focus on enhancing tissue retention to improve drug efficacy. The KasA inhibitor indazole sulfonamide DG167 (GSK3011724A) (Figure 3 and Figure 7) exhibits potent activity with a minimum inhibitory concentration (MIC) of 0.39 μM, and shows synergy with INH, enhancing antimicrobial efficacy and potentially targeting persistent *M. tuberculosis* populations [114]. Other KasA-targeting compounds, such as the indazole derivative JSF-3285 [115], further potentiate anti-tubercular activity when combined with INH or rifampicin (RIF). The chemical structures of DG167, JSF-3285, and TLM are shown in Figure 7A.

#### 5.1.3. Anti-Tuberculosis Drugs Targeting β-Hydroxyacyl-ACP Dehydratases

In *M. tuberculosis*, the β-hydroxyacyl-ACP dehydratases—HadA, HadB, HadC, and HadD—catalyze the dehydration of β-hydroxyacyl-ACP intermediates to trans-2-enoyl-ACP, facilitating meromycolic chain elongation. HadAB and HadBC are heterodimeric enzymes, consisting of HadA, HadB, and HadC subunits encoded within a single gene cluster [116,117]. Functionally, HadAB catalyzes the early elongation cycles of the FAS-II system, contributing to all types of MA biosynthesis, while HadBC and HadBD act in later elongation cycles, particularly for longer-chain methoxy- and keto-MA [42,43]. While HadD, an enzyme recently identified by Lefebvre et al., is encoded separately, playing a crucial role in FA elongation and is universally present in all sequenced *Mycobacterium* genomes [62].

The phenylthiomethyl-[1,4]-naphthoquinone inhibitors NAS-21 and NAS-91, initially developed as antimalarial agents, also exhibit potent activity against *M. tuberculosis* and *M. bovis* BCG, specifically targeting HadB (encoded by *Rv0636*) [118,119]. NAS-21 and related analogs show increased minimum inhibitory concentration (MIC) against *M. bovis* BCG strains overexpressing the *Rv0636* gene, inhibiting α-MA and keto-MA biosynthesis in a dose-dependent manner [118]. Isoxyl (ISO) and thiacetazone (TAC), prodrugs activated by the monooxygenase EthA, are strongly implicated in targeting the β-hydroxyacyl-ACP dehydratases (HadA-C), as resistance develops upon overexpression of HadAB/BC [120,121]. Both drugs were historically used in combination with INH for tuberculosis treatment. However, ISO has been discontinued for clinical use due to poor absorption kinetics and low bioavailability [122], while TAC has seen limited clinical application due to concerns regarding its toxicity [123]. The chemical structures of NAS-21, NAS-91, ISO, and TAC are shown in Figure 7B.

#### 5.1.4. Anti-Tuberculosis Drugs Targeting the SAM-MT

*Mycobacteria* utilize the S-adenosylmethionine-dependent methyltransferases (SAM-MTs) family to modify meromycolic chains via cyclopropanation, methylation, and oxidation. Among these enzymes, PcaA catalyzes the proximal cyclopropanation of α-MA, while CmaA2 acts as the sole *trans*-cyclopropane synthase for oxygenated MA [54]. MmaA1, another SAM-MT enzyme, converts *cis*-olefins to *trans*-olefins and introduces methyl branches into hydroxy-MA [54]. Loss of MmaA2 impairs distal cyclopropanation and slightly disrupts *cis*-cyclopropanation in methoxy-MA [56]. MmaA4 methylates *cis*-double bonds and catalyzes hydroxylation in the presence of water, producing hydroxy-MA, a branching intermediate that directs the synthesis of keto-MA or methoxy-MA, with the latter mediated by MmaA3 [59]. These modifications play crucial roles in *M. tuberculosis* pathogenicity, virulence, and persistence, making SAM-MTs promising targets for novel anti-tuberculosis therapies.

Structural analogs of the cofactor S-adenosylmethionine (AdoMet), such as S-adenosyl-L-homocysteine (AdoHcy) and the antibiotic Sinefungin, inhibit SAM-MT activity by binding to MmaA4 (Figure 3 and Figure 7C) [124,125]. However, in vitro assays showed limited impact on MA biosynthesis [126]. Vaubourgeix et al. identified a more effective AdoMet analog, S-adenosyl-N-decyl-aminoethyl (SADAE) (Figure 7C), that inhibits SAM-MT activity in *M. tuberculosis* and *M. smegmatis* by occupying the cofactor binding site with its adenosyl moiety, while its C10 alkyl chain extends into the hydrophobic substrate pocket, enabling dual competitive inhibition of cofactor and substrate binding [126]. In vitro, SADAE potently inhibits SAM-MTs, including MmaA4, MmaA2, PcaA, and CmaA2, with IC_50_ values ranging from 0.1 to 1 μM. In vivo, SADAE penetrates cellular barriers, suppressing SAM-MT activity at approximately 100 μM, inhibiting *M. tuberculosis* and *M. smegmatis* growth [126]. Its multi-target inhibition reduces *M. tuberculosis* virulence and persistence, while delaying the onset of drug resistance, highlighting a new direction for developing next-generation therapeutics against drug-resistant tuberculosis. The chemical structures of AdoHcy, Sinefungin, and SADAE are shown in Figure 7B.

#### 5.1.5. Anti-Tuberculosis Drugs Targeting Polyketide Synthase 13 (Pks13)

Pks13 is a large enzyme (1733 amino acids) with five functional domains: N-terminal acyl carrier protein (N-ACP), C-terminal acyl carrier protein (C-ACP), ketosynthase (KS), acyltransferase (AT), and a bifunctional thioesterase (TE) domain [127]. The TE domain catalyzes both ester bond formation through thioester hydrolysis and acyl transfer, specifically facilitating the transfer of the antigenic molecule TMM to cell wall [128].

**Figure 8:**
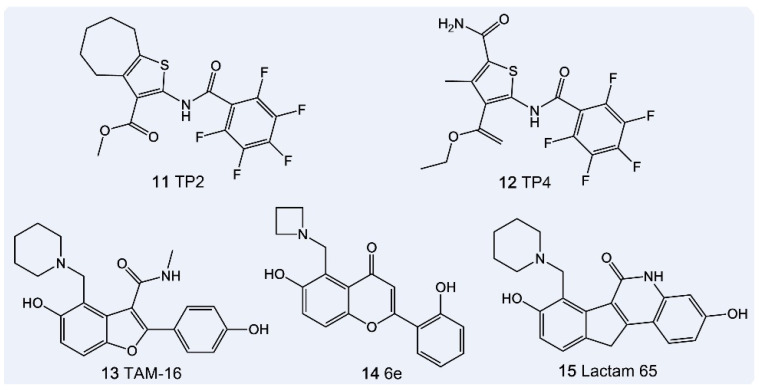
Anti-tuberculosis Drugs Targeting Pks13.

Thiophene derivatives, including TP2 and TP4, bind to the N-ACP domain of Pks13, inhibiting its enzymatic activity and disrupting MA biosynthesis, which leads to mycobacterial cell death, while also exhibiting a synergistic effect when combined with isoniazid (INH) against drug-resistant, dormant bacterial populations [129]. However, thiophene compounds are metabolically unstable due to hydrolysis by endogenous esterases. In contrast, benzofuran-based lead molecules, such as TAM16, target the TE domain and exhibit favorable drug-like properties, pharmacokinetics, and safety profiles. [130]. TAM16 has a resistance frequency approximately 100 times lower than INH and shows synergistic antibacterial effects when combined with RIF, the ATP synthase inhibitor TMC207, and streptomycin, especially against resistant strains [130]. Recently, structure-guided small molecules targeting the TE domain, including compounds 4*H*-chromen-4-one 6e (MIC = 0.45 μg/mL) [131], and 5*H*-benzofuro [3,2-*c*] quinolin-6-ones (MIC = 0.0313–0.0625 μg/mL) [132], have demonstrated promising inhibitory activity, representing a significant advancement in anti-tuberculosis drug development. The chemical structures of TP2, TP4, TAM-16, 6e, and Lactam65 are shown in Figure 8.

#### 5.1.6. Anti-Tuberculosis Drugs Targeting the Membrane Transporter Protein MmpL

The MmpL protein family, a subset of the ABC transporter superfamily, is critical for synthesizing TDM and other cell wall components by transporting the MA precursor TMM across the cell membrane [69]. The *M. tuberculosis* H37Rv genome encodes up to 14 MmpL proteins, with MmpL3, MmpL4, MmpL7, MmpL8, MmpL10, and MmpL11 playing key roles in MA biosynthesis and bacterial virulence [133]. MmpS proteins serve as periplasmic accessory factors, assisting MmpL proteins in TMM transport and maintaining MA homeostasis [134]. The *Rv0206c* gene, encoding MmpL3, is an essential lethal gene in *M. tuberculosis*. Its inactivation results in TMM accumulation and reduced TDM levels, disrupting MA biosynthesis, compromising cell wall integrity, and triggering bactericidal effects [135]. MmpL3 is regulated by TtfA, an essential transport cofactor, and *MSMEG_5308*, a stabilizing factor [136]. TtfA interacts with MmpL3 at the cell membrane, playing a critical role in the growth of *M. tuberculosis* and *M. smegmatis*, while *MSMEG_5308* stabilizes the MmpL3/TtfA heterodimer, inhibiting MmpL3-mediated transport and promoting TMM accumulation [136]. MmpL11 contributes to the transport of monomycolyl diacylglycerol (MMDAG) and wax ester-MA. Loss of MmpL11 results in the accumulation of mycocerosyl phospholipid (MycPL), impairing biofilm formation, intracellular growth, and non-replicating persistence in *M. tuberculosis* [137]. The periplasmic domains of both MmpL3 and MmpL11 interact with the lipoprotein LpqN to enhance MA transport [138].

**Figure 9:**
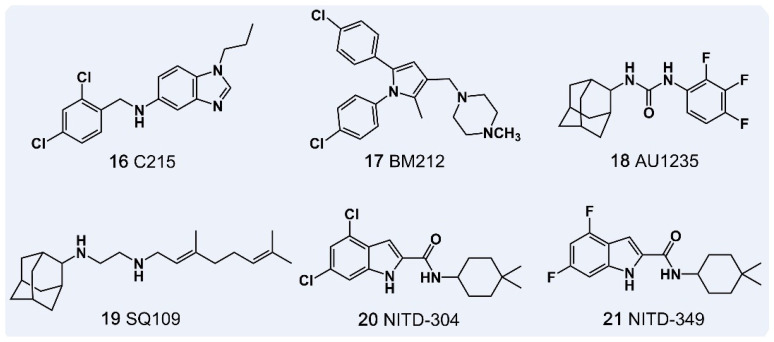
Anti-tuberculosis Drugs Targeting the Membrane Transporter Protein MmpL.

Significant advances in drug development targeting MmpL3 have been made driven by the three-dimensional structural data. Notable inhibitors include the benzimidazole C215 [139], 1,5-diphenylpyrrole BM212 and derivatives [140], adamantyl-urea AU1235 [141], ethylenediamine derivative SQ109 [142], and indole amide NITD-304 and NITD-349 [143] (Figure 9). These compounds inhibit MmpL3, thereby blocking TMM transport and disrupting MA biosynthesis. Resistance studies by McNeil et al. on spiroaminobenzothiazole compounds revealed that mutations predominantly occur in the *Rv0206c* gene encoding MmpL3, underscoring its critical role in drug resistance [141]. These mutations may alter substrate, cofactor, or stabilizer binding specificity and affinity. Future research into the resistance mechanisms of this class of compounds should focus on how these mutations influence the conformational stability of the binding pocket and the efficiency of energy coupling. A comprehensive understanding will benefit from integrating site-directed mutagenesis, protein structural analysis, and molecular dynamics simulations to elucidate the molecular basis of drug resistance. Although several MmpL3 inhibitors show potent in vitro activity against *M. tuberculosis*, clinical development is hindered by pharmacokinetic challenges, including high lipophilicity and poor solubility [144]. This paradox arises from the intrinsic nature of the target: inhibiting membrane proteins involved in lipid transport typically requires lipophilic compounds. However, high lipophilicity often compromises oral bioavailability and formulation stability. Despite these challenges, MmpL3 remains an attractive target due to its essential role in bacterial survival. Overcoming its physicochemical limitations will require innovative strategies, such as the development of hydrophilic prodrugs or nano-delivery systems, to balance affinity and solubility. While most research has focused on MmpL3, other members of the MmpL family, including MmpL5 and MmpL7, as well as accessory proteins like MmpS, TtfA, and *MSMEG_5308*, play crucial roles in MA biosynthesis. Further exploration of therapeutics targeting these additional MmpL proteins and their cofactors is essential for expanding MmpL-based anti-tuberculosis strategies.

#### 5.1.7. Additional MA Inhibitors

**Figure 10:**
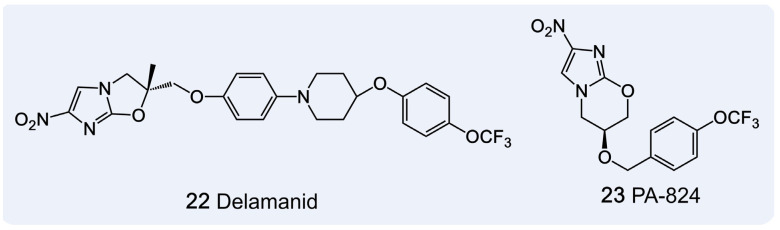
Two MA Inhibitors with Unknown Targets.

In addition to established anti-tuberculosis drugs, several compounds with partially understood mechanisms but significant clinical potential have attracted attention. Notably, Delamanid and Pretomanid (Figure 10) are two such agents.

Delamanid, a bicyclic nitroimidazo-oxazole compound, exerts its antibacterial effect through activation by the F420-dependent nitroreductase (Ddn) enzyme. Mechanistically, it disrupts *M. tuberculosis* cell wall integrity by inhibiting the synthesis of methoxy-MA and keto-MA [145,146]. Approved for use in multiple countries for the treatment of MDR-TB, Delamanid has shown promising efficacy in pediatric patients [147]. However, its clinical use is limited by side effects, including nausea, vomiting, headache, and QTcF interval prolongation [5]. Furthermore, mutations in the *Ddn* gene can impair drug activation, potentially leading to resistance and limiting its long-term effectiveness [148].

Pretomanid PA-824, a bicyclic nitroimidazole, is also activated by Ddn and disrupts MA biosynthesis. Additionally, upon activation, PA-824 releases nitric oxide (NO), which disrupts the bacterial electron transport chain and induces redox imbalance, particularly under anaerobic conditions, leading to respiratory toxicity [149]. A combination regimen of Pretomanid with Bedaquiline and Linezolid has been FDA-approved for treating MDR-TB and XDR-TB, showing promising clinical outcomes [150].

While Delamanid and Pretomanid offer promising treatment options for MDR-TB and XDR-TB, their diverse resistance mechanisms and potential long-term side effects necessitate further research and continuous monitoring. Such efforts are essential for optimizing personalized treatment strategies, advancing precision medicine, and identifying novel therapeutic targets in the fight against tuberculosis.

### 5.2. Metabolic Engineering Based on Key Enzymes in MA Biosynthesis

Steroidal drugs represent the second-largest class of pharmaceuticals following antibiotics [151]. Advances in their production are critical for the pharmaceutical industry. As of now, chemical synthesis remains the primary method for producing most steroidal drugs and their intermediates. Only a few products, such as 4-AD, have achieved green industrial production through microbial transformation. This limited adoption is largely due to the low conversion efficiency and selectivity of current microbial biotransformation methods. Overcoming these limitations requires the development of high-performance microbial strains combined with advanced metabolic engineering approaches. Mycobacteria possess unique capabilities for the uptake of cholesterol and phytosterols, as well as the degradation of side chains, which facilitate the targeted accumulation of key intermediates [152]. *M. neoaurum*, a non-pathogenic species, is extensively utilized in steroid biotransformation due to its industrial potential. Recent efforts have focused not only on optimizing conversion efficiency via steroid metabolic pathway engineering but also on enhancing sterol substrate uptake and product efflux by genetically modifying cell wall components, particularly those involved in MA biosynthesis [9]. This strategy aims to improve cell permeability, thereby increasing the efficiency of steroid drug production.

In 2017, Xiong et al. knocked out the *mmpL3* gene, which encodes the TMM transmembrane transporter, in *M. neoaurum* ATCC 25795. This modification increased cell permeability by 23.4%, enhanced sterol substrate consumption by 15.6%, and raised the yield of 4-HBC by 24.7% [7]. In their subsequent work, the team deleted the *kasB* gene, encoding β-ketoacyl-acyl carrier protein synthase. Altering the ratio of different MA types in the cell wall resulted in a 137.7% increase in 9-OHAD production [8]. In 2022, Xiong and colleagues again enhanced 9-OHAD production by 75.8% using a dual mutant strain by knocking out of the *fbpC3* gene, which encodes the secreted esterase Ag85, and the *embC* gene, involved in arabinogalactan assembly [9].

Yamaryo-Botte et al. demonstrated that acylation by the acyltransferase *TmaT* is essential for transporting TMM to the cell wall via MmpL3, as *TmaT* knockout in *C. glutamicum* results in TMM accumulation [153]. Palčeková et al. found that SucT, another acyltransferase, modifies arabinose structures in arabinogalactan (AG) and lipoarabinomannan (LAM) by adding succinyl groups. In *M. smegmatis*, *SucT* deletion alters the cell envelope’s hydrophobicity and rigidity without significantly affecting AG and LAM biosynthesis [154]. Chen et al. knocked out *TmaT* and *SucT* individually in *M. neoaurum* ATCC 25795. The knockout strains exhibited improved 9-OHAD conversion rates, from 45.3% to 62.4% and 67.9%, respectively. However, the combined deletion of both genes did not further enhance conversion efficiency, likely due to simultaneous disruption of both covalent and non-covalent layers of the cell wall, which caused abnormal changes in cell morphology and structure [155].

These studies highlight that modifying mycobacterial cell wall architecture, particularly genes involved in MA biosynthesis, can enhance cell permeability, thereby improving steroid biotransformation efficiency. This strategy not only links cell wall permeability with conversion efficiency but also provides molecular targets for rational engineering of microbial strains in industrial biotransformation applications.

## 6. Conclusions and Perspectives

MAs, as essential components of the mycobacterial cell wall, play pivotal roles in maintaining structural integrity, regulating cell surface hydrophobicity, controlling antibiotic permeability, and conferring drug resistance. Extensive studies have elucidated the catalytic enzymes and mechanisms involved in MA biosynthesis, including the type I (FAS-I) and type II (FAS-II) fatty acid synthase systems, as well as the MmpL family of membrane transporters. The first-line anti-tuberculosis drug isoniazid (INH) targets key enzymes within this biosynthetic pathway [97]. However, these prodrugs rely on enzymatic activation and are associated with long treatment regimens, severe adverse effects, and increasing drug resistance, highlighting the need for new therapeutic strategies. Recent efforts have focused on developing novel inhibitors that directly target essential enzymes in MA biosynthesis, and on designing dual-mechanism compounds such as prothionamide (PTH) [96] and the indazole sulfonamide DG167 [114]. These agents not only retain prodrug-like inhibitory mechanisms but also directly block critical enzyme in MA synthesis, such as DprE1 and KasA. SADAE, designed by Vaubourgeix et al., achieves synergistic inhibition by simultaneously targeting both the enzyme’s catalytic site and cofactor binding site, thereby enhancing inhibitory potency and presenting a valuable dual-site engagement design concept [126]. Machutta et al. demonstrated that TLM inhibits three key enzymes—KasA, KasB, and FabH—blocking MA synthesis. This approach underscores the viability of multi-target inhibitor strategies and presents new directions for drug development [112]. Despite their therapeutic potential, MA biosynthesis inhibitors often exhibit pharmacokinetic limitations due to high lipophilicity, including poor solubility, rapid metabolism, and nonspecific plasma protein binding. Continued structural and pharmacological optimization is therefore required to improve their drug-like properties.

Advances in omics technologies have led to the identification of new enzymes involved in MA biosynthesis, thereby driving the development of novel anti-tuberculosis agents targeting these enzymes. However, several critical metabolic mechanisms within this pathway remain unresolved. For example, the enzymatic process and key catalysts mediating the formation of α-alkyl, β-ketoacyl trehalose from α-alkyl, β-ketoacyl intermediates and trehalose are not yet defined. The enzyme CmrA, which reduces α-alkyl and β-ketoacyl trehalose to TMM [68], remains structurally uncharacterized, and its catalytic mechanism is unclear. Additionally, the oxidase responsible for converting hydroxy-MA to keto-MA has yet to be identified. Moreover, the regulation of chain elongation, double-bond introduction, post-synthetic modifications, enzyme regulatory networks, and MA transport from intracellular synthesis sites to the cell wall are still poorly understood. Clarifying these unresolved mechanisms represents a major research priority, offering potential targets for novel anti-tuberculosis therapeutics. Moreover, addressing these gaps is essential for developing safer, more effective drugs and for engineering metabolically optimized microbial strains.

Recent advances in cryo-electron microscopy (cryo-EM) and nuclear magnetic resonance (NMR) resolution, continuous optimization of X-ray crystallography, and the advent of protein structure prediction tools such as AlphaFold have enabled high-precision structural elucidation of numerous *M. tuberculosis* target proteins. These developments provide a solid structural and theoretical foundation for the rational design of new therapeutics against multidrug-resistant (MDR-TB) and pre-extensively drug-resistant tuberculosis (pre-XDR-TB). Leveraging artificial intelligence (AI)–driven drug screening and design, structure-based approaches can efficiently mine biochemical and structural databases using atomic-level target information to accurately identify key binding sites and design potent anti-tuberculosis candidates. In parallel, phenotype-based screening and whole-cell analyses can validate candidate compounds for cell wall penetration and in vivo efficacy, substantially accelerating the drug discovery process. Future anti-tuberculosis drug development is expected to follow several key directions: (1) Expanding beyond classical targets to identify novel molecular targets within critical pathways such as MA biosynthesis and regulation; (2) Designing multitarget agents to achieve synergistic antibacterial effects and delay resistance emergence; (3) Developing combination therapies integrating chemotherapeutics, immunomodulators, and targeted agents for multidimensional “target–immune–metabolic” interventions to reduce toxicity and enhance efficacy; (4) Advancing precision and personalized medicine through genomic and resistance-profile-guided therapy design, coupled with AI-driven pharmacodynamic modeling for optimized treatment strategies.

Although the engineering of sterol-metabolizing strains via modification of the MA biosynthetic pathway is still in its early stages, initial progress has been made. Current research primarily investigates the effects of *M. neoaurum* genes—such as *MmpL*, *KasB*, and those involved in lipoarabinomannan assembly—on the biotransformation efficiency of steroid intermediates including 4-AD and 9-OHAD [7,8,155]. Future studies could target additional key enzymes in the MA biosynthetic pathway to fine-tune the composition and ratio of MA species in the cell wall, thereby modulating cell wall permeability and enhancing the catalytic performance of *Mycobacterium*-based chassis strains. This strategy would provide a technological foundation for efficient and sustainable steroid drug production. Furthermore, this approach offers a framework for improving the broad-spectrum performance of engineered strains. Expanding this research to other pathways influencing cell permeability and implementing multidimensional metabolic engineering could further support industrial-scale applications.

## Data Availability

No datasets were generated or analyzed during the current study.
